# Binaural Processing Deficits in Autism Spectrum Disorder

**DOI:** 10.3390/audiolres16020034

**Published:** 2026-02-27

**Authors:** John A. Kara, Tashonda B. Vaughn, Tanya Gandhi, Charles C. Lee

**Affiliations:** Department of Comparative Biomedical Sciences, School of Veterinary Medicine, Louisiana State University, Baton Rouge, LA 70803, USAgandhi.tanya11@gmail.com (T.G.)

**Keywords:** auditory, interaural, neural circuits, behavior, neurophysiology, neurodevelopment, neuroanatomy

## Abstract

The central auditory system integrates signals received from both ears to derive information about the spatial and spectral features of the emitting sound source. This binaural processing of acoustic information is critical for both communication and environmental awareness. However, these binaural computations may become disrupted in individuals diagnosed with autism spectrum disorder (ASD), potentially leading to difficulties with speech perception, sound attention, and sensory hypersensitivity. Here, we present a narrative review of the emerging evidence regarding binaural processing deficits in ASD. These deficits include elevated thresholds for interaural time and level differences and reduced sound localization accuracy. In addition, physiological data suggests that these behavioral traits correspond with abnormal activity in central auditory structures. Molecular and cellular alterations to central auditory circuits may underlie these behavioral and physiological features, which could arise from both genetic and environmental factors. Overall, binaural processing alterations in ASD remain under-studied, with a need for future studies to identify neural circuit-level mechanisms and potential interventions.

## 1. Introduction

Autism spectrum disorder (ASD) represents a group of neurodevelopmental conditions that are characterized by two main criteria: restricted and repetitive interests and social communication deficits [1,2,3]. The current DSM-5 diagnostic criteria for ASD have evolved from the behaviors first described by Leo Kanner in his seminal 1943 paper, where he wrote, “these children have come into the world with innate inability to form the usual, biologically provided affective contact with people, just as other children come into the world with innate physical or intellectual handicaps,” though his description of the eleven children in his report still remains largely applicable today [4,5].

Several studies and reviews have reported the following information about ASD: (1) it represents a group of neurodevelopmental conditions that are characterized by restricted and repetitive interests and social communication deficits [1,2,3,4,5]; (2) autism was originally considered a rare disorder [6], but (3) ASD’s prevalence in the United States has grown in the past two decades from 1 in 110 to 1 in 31 children, with the world-wide prevalence currently estimated to be 1 in 127 individuals [7,8,9,10]; and (4) advances and revisions of the diagnostic questionnaire have likely impacted the increasing prevalence of ASD [11,12].

Reviews of prior investigations into the underlying characteristics noted autism-specific, sensory dysfunction in social and cognitive functioning [13,14]; individuals with ASD are atypically reactive and unable to habituate to sensory stimuli [15,16,17]. Among 43 sensory domains potentially affected in ASD, auditory processing is noted to play a particularly critical role in communication and social interactions [18,19]. Many individuals with ASD exhibit hypersensitivity to sounds, as well as difficulty understanding speech in noisy environments and in localizing sound sources, which suggest fundamental disruptions in how the brain encodes and integrates auditory spatial information [18,20,21,22,23]. To date, the neural mechanisms underlying sensory perceptual deficits in persons with ASD remain incompletely understood [24,25].

In comparison with other sensory systems, central auditory processing in the brain particularly relies on integrating different signals obtained from both ears to extract spatial and spectral cues about the location and identity of the emitting source, as reviewed in [26,27]. These computations occur through an intricate constellation of crossed connections coursing throughout the auditory brainstem, midbrain, thalamus, and cortex [28,29,30,31,32]. The neurotypical integration of this binaural information enables efficient sound localization, speech segregation in complex acoustic scenes, and the perception of auditory space [33,34]. All these processes depend on millisecond-level temporal precision and balanced excitatory/inhibitory signaling, which are highly sensitive to neurodevelopmental perturbations affecting circuit maturation and synaptic function [33,35]. Varied disruptions at each stage of the central auditory pathways can lead to critical deficits in normal auditory perception in those with ASD [36,37,38,39,40]. Indeed, recent evidence from both human and animal studies suggests that binaural auditory processing is atypical in ASD [41,42]. Psychophysical studies reveal elevated thresholds for interaural time (ITD) and level (ILD) detection, reduced accuracy in sound localization, and impaired spatial unmasking [43,44,45]. Electrophysiological and neuroimaging findings corroborate these behavioral results, demonstrating altered auditory brainstem responses, atypical cortical synchronization, and disrupted connectivity among auditory nuclei [36,46,47]. At the cellular and circuit levels, animal models of ASD exhibit abnormal inhibitory signaling, reduced temporal precision, and impaired synaptic refinement in binaural pathways [48,49,50,51,52]. Understanding how these multilevel alterations converge to produce the auditory and communicative challenges observed in ASD is of both fundamental and translational importance.

In this narrative review, we discuss the evidence for binaural processing deficits in ASD across behavioral, electrophysiological, structural, and cellular domains. To identify relevant studies, we searched all available dates in standard databases (PubMed, Web of Science, and Scopus) for publications that referenced autism terms (ASD, autism spectrum disorders, etc.) with binaural hearing terms (ITD, ILD, auditory, commissural, contralateral). In addition, we assessed the identified primary literature to determine any additional resources. From these sources, we present a narrative survey of binaural auditory processing and its potential disruption in ASD. We discuss the potential consequences of altered binaural processing in individuals with ASD, as a guide towards identifying future biomarkers and therapeutic approaches. Overall, we find that binaural processing deficits in ASD are under-studied; this requires greater attention from the auditory and ASD research communities.

## 2. Bilateral Connections in the Central Auditory System

**Figure 1:**
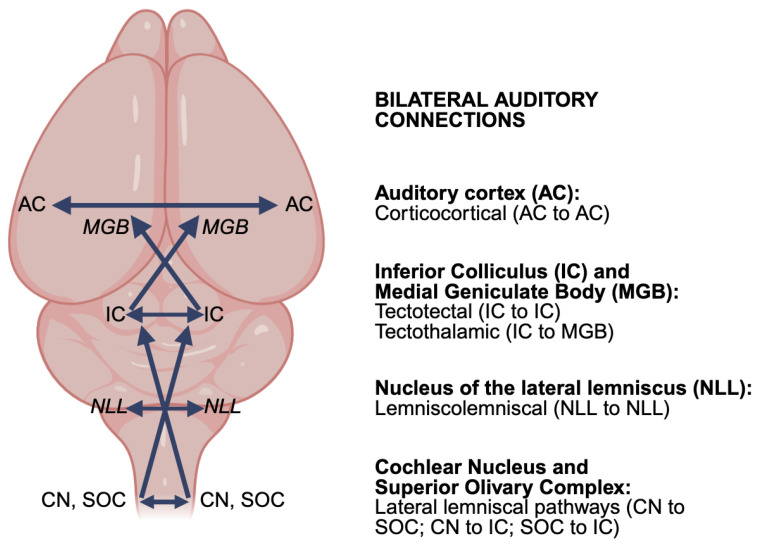
Schematic summary of major bilateral projections in the ascending auditory system. Bilateral connections occur throughout the auditory brainstem (CN: cochlear nucleus; SOC: superior olivary complex), pons (NLL: nucleus of the lateral lemniscus), midbrain and thalamus (IC: inferior colliculus; MGB: medial geniculate body), and forebrain (AC: auditory cortex). In addition, bilateral projections are also prevalent in the descending auditory system (not depicted for clarity). The schematic is of a mouse brain. Auditory structures are depicted in their general rostrocaudal and lateromedial positions, but not necessarily in their respective dorsoventral positions.

Integrating binaural sound information requires that auditory information from both ears be conveyed and combined through bilateral neural connections that cross the midline throughout the central auditory system (Figure 1) [53,54]. Much of the data regarding binaural neural circuitry is derived from a wide range of animal species, e.g., cats, rats, mice, bats, gerbils, etc. Specialized neuroethological adaptations of the basic auditory neural circuits exist across species, e.g., due to head size or communication abilities, but are summarized more generally here as they pertain to humans. Broadly, after the initial neural encoding of sound in the cochlea, auditory information is sent to the cochlear nucleus (CN) via the auditory nerve [55,56]. Thereafter, the first site of binaural convergence is the superior olivary complex (SOC) of the brainstem, which receives projections from both CNs [57,58,59]. The binaural information computed in the SOC is relayed to the nuclei of the lateral lemniscus (NLL) and integrated within the inferior colliculus (IC) in the midbrain [60,61,62]. From the IC, projections ascend to the medial geniculate body (MGB) of the thalamus and then to the auditory cortex [63,64,65,66]. Overall, the brainstem SOC is the most intensively investigated structure for the bilateral integration of auditory information, although bilateral connections are also prevalent in higher auditory structures, although their functional contributions are less clear [57,58,59,67,68].

In the brainstem, the SOC consists of several nuclei: the lateral superior olive (LSO) the medial superior olive (MSO), and the lateral (LNTB) and medial (MNTB) nuclei of the trapezoid body. These structures have well-described roles in computing differences in ILDs and ITDs, respectively [57,58,59]. In general, the LSO computes high-frequency ILDs through the integration of excitatory (glutamatergic) inputs received from ipsilateral ventral CN and inhibitory (glycinergic) inputs received from the ipsilateral MNTB, which itself receives excitatory inputs from the contralateral ventral CN [32,69,70,71]. The LSO also sends axonal projections through the contralateral lateral lemniscus (LLC) to the contralateral inferior colliculus (IC) [67,68]. In comparison, the MSO computes low-frequency ITDs through the integration of ipsilateral and contralateral excitatory inputs from both CNs and inhibitory inputs from the MNTB and LNTB, which themselves receive excitatory inputs from the ipsilateral and contralateral CNs, respectively [57,72,73,74]. The convergence of these bilateral inputs in the MSO likely enables the coincident detection of time-delayed sounds [72]. However, it should be noted that both the LSO and MSO have complete representations of frequencies (both high and low). As such, both structures have overlapping and complementary roles in processing ITDs and ILDs in the envelopes and fine structure of sounds [75,76,77].

The IC is the major auditory center in the midbrain that receives convergent input from ascending brainstem and descending cortical sources [78,79,80,81]. Both ICs are bilaterally connected with one another through the commissure of the inferior colliculus [28]. These projections are primarily topographically organized, with similar nuclear and subnuclear domains connected to homotopic targets in the contralateral IC [82]. Contralateral projections are composed of both excitatory (glutamatergic) and inhibitory (GABAergic) components [28,62,83,84,85]. However, heterotopic contralateral IC projections may arise from distinct cell types based on cellular morphology and neurochemical identity [28,62,68,86]. Functionally, the role of the contralateral IC projections is less clear, but they may be broadly involved in increasing the sensitivity and gain control of binaurally related sounds; however, the contribution of the separate excitatory and inhibitory contralateral projections to these functions remain unresolved [87,88,89].

In the thalamus, the MGB receives ascending input from both inferior colliculi, which are composed of excitatory (~80% glutamatergic) and inhibitory (~20% GABAergic) components [31,65,90,91]. The majority (~70%) of the contralateral tectothalamic projections are branches of the ipsilateral projection and largely target homotopic domains in both MGB, although it remains unclear whether these also form branches to the contralateral IC [28,62,92]. The function of the contralateral tectothalamic pathways is unresolved, but they may also enable gain control or the binding of acoustic objects for higher auditory processing [31].

At the level of the cerebral cortex, all auditory areas are interconnected by bilateral projections that cross through the corpus callosum and link homotopic areas (~90%), with a minority also connecting non-homotopic areas (~10%) [29,93,94,95,96]. The primary auditory cortex (A1) may also exhibit connectional modularity along the tonotopic axis, potentially related to binaural interaction bands [97,98,99,100]. The commissural connections primarily originate from and terminate within cortical layer 3, but in dorsal auditory areas, they also originate from layer 5 [29,54,97,101,102,103]. Although commissural projections were largely believed to be solely excitatory (glutamatergic) in nature, inhibitory (GABAergic) neurons, primarily parvalbumin-positive but possibly others, have been found to contribute substantially to the interhemispheric pathways [103]. Commissural auditory connections may enable the perceptual fusion of auditory information across the midline, which may be related to separate cortical streams for processing either the location or identity of the sound source [104,105,106]. However, these functions may also be in part related to descending corticofugal pathways that target contralateral sites in the midbrain and brainstem [107,108,109]. As such, descending contralateral cortical pathways can potentially influence binaural processing throughout the ascending auditory stream, as reviewed in [79].

Developmentally, the establishment of normal bilateral connectivity requires fine-tuned mediation by both intrinsic genetic and extrinsic environmental cues [110,111,112,113,114]. As such, binaural processing circuits undergo critical periods during which auditory experience calibrates sensitivity to interaural cues, which also depend on intrinsic cues for normal circuit maturation and synaptic refinement [110,115,116]. Disruption of these processes through genetic mutations, altered sensory experience, or neurodevelopmental disorders can lead to persistent deficits in spatial hearing and sound localization [110,111,112,113,114,115,116]. Disruption in any combination of these bilateral networks may contribute to the auditory perceptual anomalies observed in ASD [117,118,119]. The following section reviews the evidence for such disruptions across behavioral, physiological, and neuroanatomical domains in individuals with autism and in a relevant animal model.

## 3. Binaural Processing Alterations in Autism Spectrum Disorder

Hearing deficits, including binaural processing deficits, are observed in individuals with ASD, likely arising in more than half of ASD cases [120,121]. These perceptual deficits can impact normal spatial hearing by disrupting sound localization, source identification, and stream segregation [20,43,44,122,123,124,125,126,127,128,129]. These behavioral effects correlate with concomitant alterations to neuroanatomical and physiological measures that may arise from atypical development of auditory circuits (Table 1).

**Figure 2:**
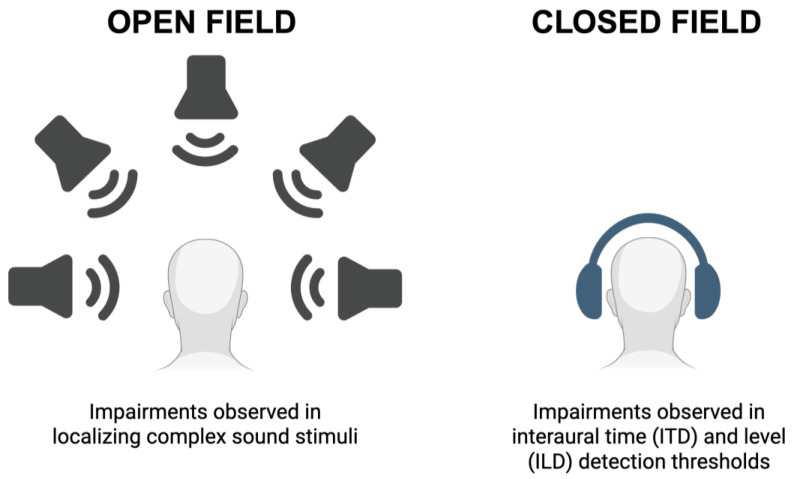
Schematic summary of binaural stimulation setups. Open-field acoustic stimuli are delivered through arrays of speakers positioned at varying horizontal and/or vertical angular displacements from the listener, while closed-field assessments are delivered directly to the listener via headphones. Individuals with ASD display varying behavioral and electrophysiological alterations in both setups.

Binaural hearing assessments can be performed in either an open- or closed-field environment (Figure 2), e.g., [41,129]. Open-field (or free-field) tasks typically involve an array of sound speakers that are situated at different horizontal and vertical displacements relative to the listener. In contrast, closed-field tasks involve sound stimuli presented directly to the listener via headphones. As with most experimental setups, each has unique advantages and disadvantages [34,130]. Open-field tests enable assessment of complex acoustic, environmental, and head-related cues, while closed-field tests enable fine control of direct aural stimulation, e.g., independent control of ITDs and ILDs. In each setup, the type and pattern of acoustic stimuli can be carefully designed to probe the spectral and temporal responsiveness of the listener. And, in the case of ASD, each type of test has implicated impairments to binaural hearing.

However, several important caveats exist regarding these studies on binaural hearing in ASD. First, it is unclear whether these studies reflect specific deficits in binaural integration, or whether they instead reflect general deficits in temporal processing (e.g., phase-locking for ITDs), or some combination of these factors. Second, non-sensory factors, e.g., attentional state or working memory conditions, are potential confounders to observed performance deficits, particularly in ASD. Finally, the broad range of experimental paradigms in these studies may yield convergent results through divergent mechanisms. As such, more direct interpretations will require further refined studies to disentangle these factors.

Overall, the severity of potential binaural hearing deficits correlates with the severity of ASD symptoms [20,123]. In many studies, ITD and ILD thresholds are elevated in individuals with ASD, although some studies indicate that only responses to ITD cues are affected in this manner (Table 1; Figure 2) [43,126,127,128]. The threshold elevations may be due to the nature of the stimuli that are presented across studies. In addition, spatial unmasking, the ability to exploit spatial separation between target and masking sounds, is reduced or impaired, which is most evident in noisy environments that require fine binaural computations to segregate auditory objects (Table 1; Figure 2) [122,126]. Based on two studies, these deficits are likely evident from early childhood, but direct comparisons across age groups have not been specifically investigated (Table 1) [126,128]. As such, it remains unclear whether potential developmental deficits in binaural hearing are malleable through early or late environmental interventions.

The neurophysiological basis for the observed deficits in binaural hearing has primarily been assessed at the cortical level, using either electroencephalography (EEG) or magnetoencephalography (MEG) [122,126,127,128,129]. These studies reveal reduced or absent auditory evoked potentials in response to binaural stimuli, which suggest that there is impaired cortical integration of binaural cues [122,126,127,128,129]. Interestingly, a recent study suggests that these deficits may encompass altered functional connectivity between frontoparietal and auditory cortices [126]. Moreover, at subcortical levels, one study suggests that the auditory brainstem response to binaural stimulation is diminished in those with ASD; which may be related to alterations in the pathways ascending past the SOC [41].

Neuroanatomical deficits have also been observed in the central auditory pathways of individuals with ASD; however, these have not been specifically assessed vis-à-vis binaural processing deficits [37,38,131,132]. For instance, a series of post-mortem studies have identified gross morphological differences (smaller overall structure and fewer, smaller neurons) in the superior olivary complex [37,38,132]. These likely correspond to similar morphological reductions in auditory-related structures observed through imaging studies, as reviewed in [131]. Further studies should assess the direct relations between these morphological, physiological, and behavioral observations.

Animal models of ASD could illuminate the underlying neural mechanisms of the binaural processing deficits observed in humans. Indeed, general auditory dysfunctions are common to many rodent models of ASD (genetic and environmental) [49,51]. These mouse models exhibit the typical panoply of neural deficits that may underlie binaural processing abnormalities, e.g., impaired synaptic development, disrupted myelination, altered long-range and local connectivity, unbalanced excitatory/inhibitory signaling, and impaired temporal synchrony [49,51]. However, as with human studies, these varied neuroanatomical and physiological changes have not been directly linked to binaural processing deficits in these animal models of ASD. A notable exception is a series of studies in a mouse model of Fragile X Syndrome (FXS), which is the most common single-gene condition that is comorbid with autism [133]. In this FXS mouse model, the binaural interaction component of the ABR exhibits increased latency at 0 ITD [134,135], which is similar, though not identical, to that observed in humans [41]. However, an important caveat with any rodent model of binaural hearing is their smaller head size, which results in preferential use of ILD cues, rather than ITD cues [41,134,135].

Overall, human and animal studies suggest that binaural processing deficits in ASD likely arise across multiple stages of the auditory pathway. These alterations can disrupt the precise temporal coding required for binaural computations in the central auditory system, which contribute to the communication challenges that define autism. A direction for future studies is to specify the precise contributions of alterations to the central auditory pathways that result in binaural hearing deficits.

## 4. Developmental Impact of Binaural Processing Deficits

How might binaural processing deficits contribute to ASD-related behaviors? Binaural cues are important for isolating sound sources in noisy environments, where competing sounds must be spatially segregated [27,105,136]. In such complex acoustic environments, individuals with ASD may become overwhelmed and have difficulty focusing on specific sound sources; the inability to spatially segregate sound sources can affect speech intelligibility, potentially leading to some communication deficits [20,23]. Moreover, deficits in binaural hearing (and hearing generally) can result in atypical language development [137,138,139]. During early development, exposure to clear speech signals is essential for the acquisition of normal rhythmic and intonation patterns, which often convey sub-textual emotional and social meaning that may be impaired in ASD [140,141,142]. Similarly, binaural hearing deficits may disrupt social orienting, which is the ability to direct attention toward salient social acoustic signals; this can also impair the navigation of complex social interactions [143,144,145].

Auditory hypersensitivity is another common feature in ASD, which may also be impacted by binaural processing deficits [105,146,147]. The reduced ability to localize or segregate sound sources could potentially increase the perceived intensity and ambiguity of auditory stimuli [136,148,149]. More generally, inaccurate filtering of spatial cues may result in sound being experienced as intrusive and unpredictable. At the neural level, the central auditory system has direct connections with the amygdala, originating as early as the MGB [150]. As such, a heightened auditory neural response could aberrantly engage these emotional circuits to reinforce avoidance behaviors and anxiety in noisy environments.

Finally, multisensory integration and attentional control could be impacted by altered binaural processing in ASD [151,152,153]. Individuals with ASD often exhibit impaired audiovisual integration, especially in noisy environments; the alignment of auditory and visual maps could be affected by binaural processing deficits, leading to difficulties in audiovisual speech perception [154]. This may impose greater demands on working memory and attention during communication. The need to rely more heavily on other cues to compensate for degraded auditory input could increase cognitive load, contributing to fatigue and social withdrawal. These effects may be especially pronounced in children, whose developing neural systems are more vulnerable to the developmental consequences of early binaural processing deficiencies [20,120,125].

## 5. Conclusions

Binaural processing deficits are an under-studied facet of ASD, which likely contribute to its primary and secondary behavioral symptoms. These abnormalities in binaural auditory processing can impact communication, attention, and sensory regulation in ASD. The inability to accurately localize and segregate sounds undermines speech comprehension and social interaction. Moreover, the distorted auditory spatial representations could contribute to sensory overload and anxiety.

Given the emerging evidence, binaural-specific assessments of behavior and physiology may be warranted for persons with ASD. A standard battery of assessments could include the testing of: contralateral acoustic reflexes, contralateral suppression OAEs, dichotic listening, masking level differences, and others. The implementation of such assessments could result in improved treatments and expand our understanding of the magnitude and directionality of binaural processing deficits across the spectrum of these conditions. Finally, future studies should expand on the fundamental mechanisms governing how these binaural perceptual deficits arise, in order to develop effective interventions and treatments.

## Figures and Tables

**Table 1:** Evidence for binaural processing deficits in individuals with ASD.

Study	Results
Teder-Sälejärvi (2005) Ref. [122]	Behavior: Males with ASD (29–39 years old) exhibited a reduction in performance in identifying a sound source in a noisy open-field environment, although performance was unaffected in a simple localization task. EEG: Individuals with ASD exhibited a more shallow fall-off of the N1 auditory event-related potentials in the noisy sound localization task.
Visser et al. (2013) Ref. [44]	Behavior: Adults with ASD (~27 years old) exhibited worse performance in localizing sound in the vertical but not the horizontal domain in an open field. In addition, individuals with ASD exhibited shorter temporal binding windows for the precedence effect.
Brock et al. (2013) Ref. [128]	MEG: Boys with ASD (8–11 years old) lacked an object-related negativity response in both hemispheres and exhibited increased differential responses in the right hemisphere to pitch stimuli, when presented with a dichotic stimulus with ITD-related cues.
Lodhia et al. (2014) Ref. [129]	EEG: Adults with ASD (~22 years old) exhibited a reduction in the object-related negativity response, but no change in the P400 response, when presented with a dichotic stimulus with ITD-related cues.
Skewes and Gebauer (2016) Ref. [123]	Behavior: Adults with ASD (~27 years old) exhibited poorer performance in a horizontal sound localization task, i.e., pure-tone, sound intensity panned between left and right headphones. Performance of individuals with ASD was ‘more sub-optimal’ than that of controls, based on a Bayesian signal-detection model.
Soskey et al. (2017) Ref. [20]	Behavior: Youths with ASD (10–17 years old) exhibited worse performance on focusing on sound cues originating from in front of them in an open field. Performance deficits were correlated with severity of ASD diagnoses.
Lodhia et al. (2018) Ref. [127]	Behavior: Adults with ASD (~25 years old) exhibited no difference in auditory object formation based on dichotic pitch stimuli delivered through headphones. EEG: Object-related negativity response was absent for ITD-related cues, but not ILD-related cues.
ElMoazen et al. (2020) Ref. [41]	ABR: Children with ASD (years old) exhibited decreases in the predicted amplitude of the binaural interaction component of the auditory brainstem response, between peaks IV and VI.
Fujihira et al. (2022) Ref. [43]	Behavior: Adults with ASD (20–45 years old) exhibited higher threshold sensitivity to ITD and ILD cues presented through headphones. Sensitivity to ITD cues varied across a greater range, while ILD sensitivity was not as diversely distributed.
Osorio et al. (2025) Ref. [126]	MEG: Children with ASD (6–17 years old) exhibited reduced auditory cortical activation and altered functional connectivity between frontoparietal and auditory cortices, in an ITD swapping ‘jump’ stimulus presented through headphones.

## Data Availability

No new data were created or analyzed in this study.

## Abbreviations

The following abbreviations are used in this manuscript:

A1	Primary auditory cortex
ASD	Autism spectrum disorder
CN	Cochlear nucleus
IC	Inferior colliculus
ILD	Interaural level difference
ITD	Interaural time difference
LNTB	Lateral nucleus of the trapezoid body
LSO	Lateral superior olivary complex
MGB	Medial geniculate body
MNTB	Medial nucleus of the trapezoid body
MSO	Medial superior olivary complex
SOC	Superior olivary complex
